# Single-feature polymorphism discovery in the barley transcriptome

**DOI:** 10.1186/gb-2005-6-6-r54

**Published:** 2005-05-11

**Authors:** Nils Rostoks, Justin O Borevitz, Peter E Hedley, Joanne Russell, Sharon Mudie, Jenny Morris, Linda Cardle, David F Marshall, Robbie Waugh

**Affiliations:** 1 Scottish Crop Research Institute, Genome Dynamics, Invergowrie, Dundee, DD2 5DA, Scotland, UK; 2 University of Chicago, Department of Ecology and Evolution, Chicago, IL 60637, USA

## Abstract

A probe-level model for analysis of GeneChip gene-expression data is presented which identified more than 10,000 single-feature polymorphisms (SFP) between two barley genotypes. The method has good sensitivity, as 67% of known single-nucleotide polymorphisms (SNP) were called as SFPs. This method is applicable to all oligonucleotide microarray data, accounts for SNP effects in gene-expression data and represents an efficient and versatile approach for highly parallel marker identification in large genomes.

## Abstract 2

A probe level model for analysis of GeneChip gene expression data is presented which identified more than 10,000 single-feature polymorphisms between two barley genotypes, with a high sensitivity. This method is applicable to all oligonucleotide microarray data.

## Background

Whole-genome sequences of *Arabidopsis *and rice have provided a fundamental platform for the discovery of gene content and function in dicot and monocot plants. Research on the model species has provided a wealth of knowledge on universal biochemical and genetic processes, as well as the development of analytical tools that are applicable to other plant species [1-3].

The availability of abundant, high-throughput sequence-based markers is the key for detailed genome-wide trait analysis. Single-nucleotide polymorphisms (SNP) are the most common sequence variation and a significant amount of effort has been invested in resequencing alleles to discovery SNPs. In fully sequenced small-genome model organisms SNP discovery is relatively straightforward, although high-throughput SNP discovery in natural populations remains both expensive and time-consuming [4].

A number of recent studies have reported the use of oligonucleotide arrays, including expression arrays, for SNP detection in a highly parallel manner [5]. In these studies, whole genomic DNA was demonstrated to work very well for simple organisms such as yeast [6,7], and even complex, albeit relatively small genomes, such as *Arabidopsis *[8]. However, the application of oligonucleotide arrays for SNP detection in large genomes, such as human, has relied on prior complexity reduction using PCR-based enrichment [9,10]. The use of oligonucleotide arrays for simultaneous genotyping and gene-expression analysis using RNA target has also been reported in yeast [11]. While there is arguably little need for enhanced SNP discovery in yeast, the real power of the approach came from coupling genotyping and gene expression analysis.

For large-genome species, including crops such as wheat and barley, full-genome sequences may not be available in the near future. This has been compensated to some extent by model species that have allowed conserved biological processes to be studied. However, while *Arabidopsis *and rice provide insights into universal genetic, structural and developmental processes, they fail to address many topics relevant to crop-plant species, such as yield, yield stability and quality. Rice has a long history as a genetic model that has been strengthened by release of draft genome sequences [12,13]. As a result of conservation of synteny at the genomic level it has been promoted as a model for the grasses [14]. However, unlike the temperate cereals such as wheat and barley, rice cultivation occurs under short days and rather specific environmental conditions, its end uses are distinct and numerous exceptions to conserved synteny have now emerged [15-17]. Together, these highlight the limitations of rice as a universal genetic model for the cereal grasses.

Wheat and barley together constitute one third of world cereal production [18]. Barley in particular is cultivated throughout the world, in environments as diverse as arctic regions of Northern Europe, subtropical regions of Africa and the highlands of the Andes and the Himalayas [19]. Barley breeding has created varieties tailored mainly for animal feed, malt production and human food [20]. Ultimately, environmental and agronomical variation is based on genetic (sequence) diversity of the barley genome, with expression of agronomic traits closely linked to environmental adaptability.

With genome sizes of around 5,200 megabase pairs (Mbp) for barley [21,22] and around 16,100 Mbp for bread wheat [21] and genomic structure consisting of gene islands interspersed with highly repetitive retrotransposon sequences [15,23], access to sequence-based markers is currently provided through highly developed expressed sequence tag (EST) resources [24].

The most important traits in crop species are generally polygenic. These have traditionally been studied using biparental mapping populations and a large pool of mapped restriction fragment length polymorphism (RFLP) and/or simple sequence repeat (SSR) markers [25]. However, with the strong trend towards genome-wide association analyses based on linkage disequilibrium (LD) [26,27] there is a clear need for robust high-density and high-throughput markers that can be effectively deployed, often in closely related elite germplasm. While the number and distribution of markers for LD studies in barley remains to be empirically determined, SNP markers offer both the sequence specificity and throughput necessary for the success of this approach. SNP discovery in large-genome species is currently limited to identifying SNPs *in silico *in EST assemblies and resequencing of EST-derived unigenes in relevant germplasm [27], and scaling-up such approaches requires significant investment of both time and funding [28-30]. An approach that would allow parallel screening of the whole 'gene space' for SNPs is therefore highly desirable.

An Affymetrix GeneChip that allows simultaneous expression analysis of 22,000 transcripts has recently become available for barley [31]. Transcription provides a native mechanism for the enrichment of gene sequences. Polymorphisms present in DNA are transcribed into the messenger RNA and can potentially affect the hybridization to the GeneChip probes, if present in a region complementary to the probe. Polymorphisms generated during mRNA processing, such as alternative splicing and polyadenylation, could also affect hybridization of the target RNA.

Here we report the use of the Affymetrix Barley1 GeneChip to identify single-feature polymorphisms (SFP), which include not only SNPs but also the processing polymorphisms mentioned above, in barley transcript profiling data from cultivars Morex and Golden Promise. The statistical algorithm presented here allowed us to distinguish genotype-dependent hybridization differences at the probe level once overall gene-expression level was accounted for, leading to the identification of 10,504 SFPs.

## Results

### Identification of SFP in Barley1 GeneChip transcription-profiling data

Gene-expression data for barley cultivars Morex and Golden Promise was generated within an international collaborative project of barley researchers (unpublished results, see Acknowledgements) and consisted of 36 GeneChip hybridizations (three replicates of six tissue types) for two genotypes. Raw microarray data are available from ArrayExpress [32,33], BarleyBase [34] and [35]. The analysis code, lists of RNA and genomic SFPs, primer sequences, and the SFP sequence confirmation table are available from our website as supplementary information [35]. The hybridization intensities for each of the perfect match (PM) probes were extracted from the .CEL files. Background correction and quantile normalization was performed using the Bioconductor package RMA [36,37]. The resulting data matrix of 22,801 probe sets with 11 PM probes each was analyzed using probe-level linear models that accounted for main fixed effects of genotype, tissue, and individual probe intensity, as well as tissue-specific differences across genotypes. One replicate from a single tissue sample of Golden Promise consistently clustered with the analogous Morex replicates and this sample was reclassified as Morex. The residuals from the linear model were saved into a matrix of 250,811 probes by 36 arrays and subsequently fitted for a genotype effect at the probe level to identify SFPs between the 17 Golden Promise and 19 Morex arrays. The Bioconductor package siggenes [37] was used to determine SFPs according to statistical analysis of microarrays (SAM) [38,39].

**Figure 1:**
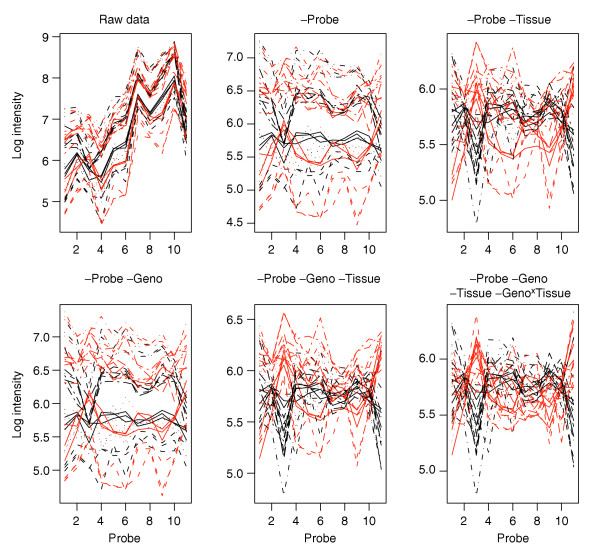
Normalization of hybridization intensity profile of 25mer probes in a probe set. The *y *axis is background-corrected normalized log intensity and the *x*-axis shows the positions of the 11 features along the unigene. Black lines trace the Golden Promise arrays, while red trace the Morex arrays. Different line types differentiate tissues. Each panel illustrates normalization for one of the major sources of variation: probe effect; probe and tissue; probe and genotype; probe, genotype and tissue; probe, genotype, tissue and genotype by tissue. 100 such plots are available from [35].

**Figure 2:**
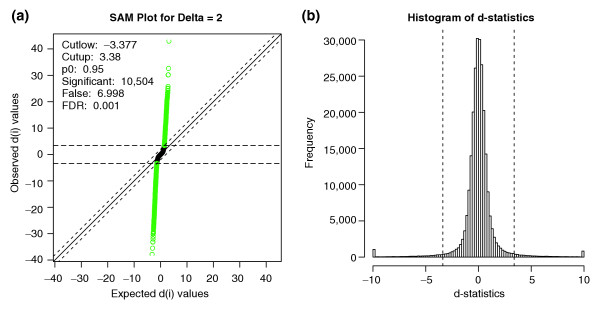
Distribution of single-feature polymorphisms. **(a) **The observed d-statistics (*y*-axis) is plotted against the expected d-statistics (*x*-axis) as determined by permutations. 10,504 significant SFPs exceeding the threshold of 0.1% FDR are shown in green. **(b**) Histogram of d-statistics truncated at ± 10. Positive scores above the threshold 3.38 are Golden Promise SFPs, and negative scores below -3.37 are Morex SFPs.

Figure 1 shows effects of the normalization steps on the expression profile of the probe set Contig10034_at and identification of a SFP in the probe 3 by removing probe and tissue effects. The large number of replicates for each genotype and the reduced genome complexity of the transcribed RNA allowed 10,504 SFPs to be identified at less than 0.1% false discovery rate (FDR) (Table 1). These SFPs resided in 3,734 Affymetrix probe sets, with one quarter of probe sets containing four or more SFPs. The magnitude of the d-statistic indicated the likelihood of a probe being called an SFP, while the sign indicated which genotype was polymorphic with regard to the reference 25mer probe on the array. Positive values predicted an SFP in Golden Promise, while negative values indicated an SFP in Morex (a complete list of SFP probes and corresponding d-statistics are available from [35]). Figure 2a shows the distribution of observed d-statistics (*y*-axis) of all probes on the array against the expected mean permutation null distribution (*x*-axis). Probes exceeding the threshold of less than 0.1% FDR, and thus containing SFP, are shown in green. Figure 2b is a histogram of the distribution of d-statistics truncated at ± 10 with thresholds shown. Figure 2b is a histogram of d-statistics truncated at ± 10 with Golden Promise SFPs in the right tail and Morex SFPs in the left tail.

### Sequence confirmation of SFP

Confirmation of SFP was done by comparison with three barley sequence datasets. Barley EST [40] is EST unigene assembly 21 [40] and contained 234 contigs with 624 predicted SFP probes where both Morex and Golden Pomise sequence were available. These were examined manually to identify SNP that overlapped 25mers on the array (see SFP confirmation table in [35] (EST dataset)).

The second set is an experimental cDNA sequence set targeting regions with predicted SFPs. Comparative DNA sequence was generated from each genotype by targeted resequencing of reverse-transcription PCR (RT-PCR) products covering 262 probes. For each genotype we combined an equal amount of RNA from all six tissue types used for hybridization to the GeneChips and converted it to a single-stranded cDNA. PCR amplification and subsequent sequencing allowed us to obtain good-quality sequence from both genotypes (see SFP confirmation table in [35] (targeted dataset)).

The third set was an experimental random genomic DNA sequence set used as a tool for SNP discovery in barley [30]. This dataset (SFP confirmation table in [35] (random dataset)) consisted of barley unigenes that had been resequenced from genomic DNA from eight barley lines, including Morex and Golden Promise, within an ongoing SNP discovery project [30]. The selection of these genes was considered random with respect to the genes predicted to have SFP. The SNP discovery project targeted the 3' ends of unigenes, the region also selected for Affymetrix probe design. The random-sequencing dataset consisted of sequences for 300 unigene contigs and covered a total of 2,204 Affymetrix probes with high-quality sequences from both genotypes.

In total, 2,699 probes were analyzed in the three datasets, of which 2,667 were unique and 31 were present in multiple datasets. Sixty-six probes were polymorphic compared to both genotypes and, since they could not be detected by our algorithm, they were excluded from further analysis. 401 unique probes contained sequence polymorphisms - 223 features were polymorphic compared to Golden Promise and 178 to Morex. 2,200 probes did not have a sequence polymorphism (Table 2; SFP confirmation table in the supplementary information at [35]).

The sequence polymorphism information was compared with the expression SFP genotype calls. Of the 401 known sequence polymorphisms, 270 were correctly predicted by our analysis, indicating 67% sensitivity. Only 25 SFPs were called where sequence confirmation revealed the polymorphism in an opposite genotype, while 155 known SNPs escaped detection. How many of the 10,504 predicted SFPs were found actually to contain a sequence polymorphism? We have sequence information for 450 of these probes, of which 270 contained SNP in the predicted genotype. This suggested that up to 40% of the 10,504 predicted SFPs may be 'falsely discovered' sequence polymorphisms (Tables 2, 3). The large discrepancy between the permutation FDR threshold of 0.1% and that determined by sequencing is due to several factors. Expression polymorphisms, such as alternative splicing or polyadenylation, do not affect primary sequence, and are also detected in our statistical model. Genes with multiple adjacent SFPs may fall into this category. In addition, true SNPs near the 25mer may be identified as SFPs due to labeling polymorphisms.

**Figure 3:**
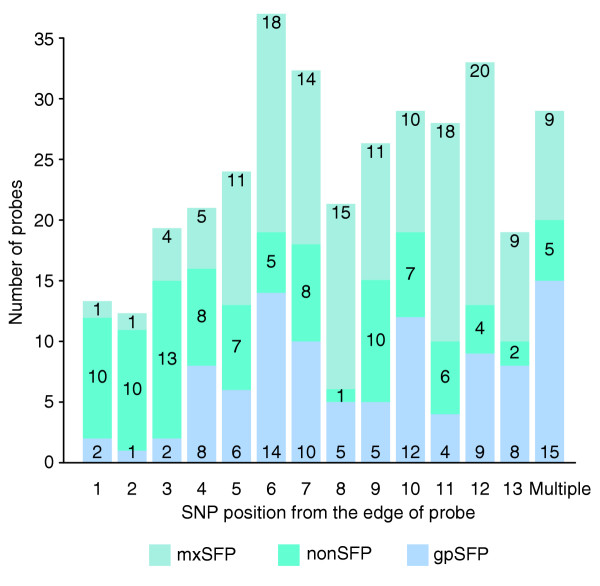
Effect of SNP position on SFP identification. The positions of the SNP in 25mers are shown on the *x*-axis as distance from the edge in nucleotides (1 - 13 nucleotides). Multiple SNP category is provided separately by a single column. The *y*-axis indicates total number of probes identified for each SNP position. Each bar is divided into the SFP categories - mxSFP, nonSFP and gpSFP (see Table 2), and shows that more accurate SFP identification is made for SNPs that reside at internal sites. The number of 25mers in each category is shown within the bars.

The ability to detect sequence polymorphisms in the RNA-profiling data depends on several properties, including the expression status of the gene in a particular tissue type, the location of the SNP within the 25mer and the hybridization properties of the particular feature. We further investigated the effect of SNP position on the ability to identify a sequence polymorphism as an SFP in transcription data. SNP position was recorded as distance from the edge of the probe, position 1 being either end and 13 being the middle of the 25mer. Figure 3 shows that, as expected, when a SNP was located in the central region (positions 6-13) it was more often called as a SFP. SNP residing in the flanking three nucleotides were called at near the background rate. Probes containing multiple SNPs were also efficiently predicted (Figure 3). A similar pattern has been seen in genomic DNA hybridizations in *Arabidopsis *[8] and yeast [6], and in RNA hybridizations in yeast [11].

### Comparison of SFP prediction in individual tissues against the full sample

We tested the sensitivity and false SNP discovery rates of our analysis with single tissue/genotype comparisons to observe how it would perform in smaller experiments. Datasets containing three replicates per genotype for each tissue type were analyzed at the threshold that again identified 10,504 SFP. In general there was a 4-16% decrease in sensitivity of the SFP prediction, which was the expected result of reducing power. On the other hand, SFP prediction in a single tissue type decreased the false SNP discovery rate by 4-5%. This was probably due to the reduction of probe-level variation in expression across tissues. In all, more than 10,000 SFP could be reliably identified even when expression profiles of single tissues were analyzed.

### Genomic DNA hybridizations

To assess the feasibility of SFP identification from barley total genomic DNA (around 5200 Mbp) [21,22], we labeled and hybridized three replicates of three highly polymorphic genotypes, Oregon Wolfe Barley Dominant and Oregon Wolfe Barley Recessive [41], and wild barley species *Hordeum vulgare *ssp. *spontaneum *(accession Mehola), to the same Affymetrix Barley1GeneChip expression array. Raw microarray data are available from [35]. Raw .CEL files were background corrected and quantile normalized and the package siggenes [37,38] was subsequently used to identify probes showing significant hybridization differences between genotypes. To assess significance, 100 random permutations were performed, FDRs were evaluated at different thresholds (Table 1) and 1,090 SFPs were identified at a 22% FDR. Although there was less power to identify SFP with nine replicates in the genomic DNA dataset compared to 36 replicates in the RNA dataset, there was also much more noise relative to signal from barley genomic DNA. This was most probably due to the complexity of the large barley genome and a lower proportion of gene regions in the labeled DNA. However, if SFPs identified in genomic DNA were real, common polymorphisms in barley should be identified by both RNA and DNA approaches, even though different genotypes were used. As shown in Table 4, a significant overlap was identified between the two SFP sets, with 114 SFPs in common where only 46 are expected by chance (*p *< 3.863e-25). More replicates and alternative gene-specific labeling conditions should improve genomic DNA SFP identification from organisms with very large genomes [9].

## Discussion

Affymetrix GeneChips designed for gene-expression analysis can be utilized for genome-wide identification of sequence polymorphisms [5]. Whole-genome DNA has been used as a hybridization target in yeast [6,7] and in *Arabidopsis *[8] to identify SFPs using expression arrays. While such an approach was valid in yeast and a small-genome model plant, the transfer of this approach to cereal crop plants with up to 100-fold larger genome sizes is problematic. The number of genes in barley is likely to be comparable to the estimated number of genes in *Arabidopsis *and rice [42,43]. However, the amount of repetitive DNA in barley will dilute the gene-specific signal in the target labelled DNA.

Until now, PCR-based artificial enrichment for a subset of sequences has been used to tackle the complexity of large genomes [10,9,44]. Using RNA as a hybridization target provides a natural way of enriching for gene sequences while maintaining all the sequence diversity present in transcribed sequences. However, sequence polymorphism effects on hybridization are concealed within the overall variation in gene-expression levels and tissue-dependent and genotype-dependent differential gene expression. Additional complexity comes from posttranscriptional sequence polymorphisms, such as alternative splicing and alternative polyadenylation. New array designs that tile probes across genes and intergenic regions will help unravel this complexity as nucleotide polymorphisms may affect single features while alternative transcripts may more often affect adjacent features.

We present here a statistical approach that allows us to reliably discern the probe-level differential hybridization between two genotypes that is often caused by sequence polymorphisms once variation in overall gene-expression level is normalized. Our approach allows the use of expression array data generated from different tissue types, and thus increases its versatility and applicability to the wide range of currently available oligonucleotide microarray data.

The analysis algorithm was applied to gene-expression microarray data generated from two barley genotypes with six tissue types each for a total of 36 array hybridizations. At a stringent 0.1% FDR, 10,504 SFPs were identified. Comparison to the available sequence-verified SNP data suggested that 67% of the known SNPs were predicted, confirming a good sensitivity. Approximately 40% of the SFP probes that were sequence-verified did not reveal any polymorphisms at the sequence level; thus, the FDR was up to 13-fold higher compared to the rate for *Arabidopsis *genomic DNA hybridizations [8]. The higher false-positive rates can be at least partly explained by variation in mRNA structure (for example, alternative splicing and polyadenylation) between tissues, and possibly between genotypes, which would lead to differential hybridization to probes but could not be detected by sequencing. A recent study using an EST collection concluded that at least 4% of barley genes may undergo alternative splicing [45]; however, more experimental data may be required to correctly model the rate of probe level variation in plant gene-expression data.

For practical application the balance between the cost of replicates and the number of replicates necessary to maintain sensitivity is important. We therefore analyzed the microarray data comparing just three replicates of each tissue type from the two genotypes (Table 3). Overall sensitivity decreased, but remained above 50%. Remarkably, the false SNP discovery rate was better for single tissue comparisons, probably because variation in mRNA transcript processing among tissues was eliminated.

Certain molecular marker applications require the precise nature of sequence changes to be known. The conventional approach to SNP discovery is based on resequencing alleles, which is particularly inefficient if the polymorphism levels are low. Prescreening for polymorphisms using, for example, single-strand conformation polymorphism (SSCP) [46] or Eco-TILLING [47], allows a reduction in sequencing costs, but these approaches are time-consuming, relatively expensive and rely on PCR. SFP detection in gene-expression microarray data allows parallel screening of a large proportion of all the organisms' gene space in one experiment. The stringency of SFP calls can also be adjusted for a particular application, that is, decreasing stringency will result in additional calls at the expense of higher false-positive rates.

Gene-expression levels are currently being treated as quantitative traits and transcript abundance variation is being mapped as quantitative trait loci (QTL) [48,49]. Incorporating SFP effects into calculations will improve accuracy of gene-expression studies and will facilitate correct assessment of allele-specific gene-expression differences. Furthermore, an SFP identified in a coding region of a gene that is differentially expressed in an allele-specific manner represents a marker linked to the regulatory regions of the gene, and as such may help distinguish between *cis *and *trans *effects in allele-specific gene expression [50-52].

## Materials and methods

### Affymetrix Barley1 GeneChip data

Affymetrix Barley1 GeneChip data was produced within an international collaborative project (A. Druka, G. Muehlbauer, I. Druka, R. Caldo, U. Baumann, N. Rostoks, A. Schreiber, R. Wise, T. Close, A. Kleinhofs, *et al*., unpublished work). Six tissue types were analyzed from two genotypes, Golden Promise (GP) and Morex (MX), with three type I replicates for a total of 36 arrays. We found that the GP genotype of one particular tissue replicate had a very high correlation with the three replicates from the comparable tissue from the MX genotype. We therefore re-assigned that replicate as genotype MX.

Genomic DNA from the wild barley *Hordeum vulgare *ssp. *spontaneum *(accession Mehola; arrays 1-3) and two morphologically diverse lines Oregon Wolfe Barley Recessive (arrays 4-6) and Oregon Wolfe Barley Dominant (arrays 7-9) [41] were prepared according to [53] and hybridized to the Affymetrix Barley1 GeneChip in triplicate according to standard methods for RNA.

### SFP prediction in gene expression data

Raw .CEL files were background corrected and quantile normalized according to Bolstad *et al*. [36]. Subsequently, only the 11 Perfect Match (PM) features from each of 22,801 probe sets were fit with the following linear model

log(*Y*tgrp) = *u *+ tissue + genotype + genotype × tissue + probe + error,

where *Y *is the background corrected normalized intensity of t (tissue), g (genotype), r (replicate), and p (probe) in a probe set. *u *is the mean probe intensity, while tissue has six states, and genotype has two states. The genotype by tissue effect accounted for tissue specific effects dependent on genotype. The residuals (22,801 probe sets × 11 probes = 250,811) from this model were fitted for a genotype effect at the probe level to reveal SFP using the Bioconductor package siggenes [37,36]. False discovery rates were estimated according to SAM [38,39] by performing 500 random permutations for RNA analysis or 100 permutations for genomic DNA analysis. The expected proportion of significantly different features (p0) was set to 0.95.

### SFP confirmation by SNP analysis *in silico*

The EST unigene assembly 21 [40] that was used to produce the Affymetrix Barley1 GeneChip [31] contains 349,709 ESTs, of which 52,556 were derived from Morex (11 libraries) and 7,439 from Golden Promise (1 library). Library details are available from the HarvEST EST database [40]. HarvEST was used to identify a total of 1,758 unigene contigs containing both Morex and Golden Promise EST.

### SFP confirmation by sequencing

192 primer pairs for 188 contigs were designed using Primer3 software [54] targeting 262 probes. Primers were supplied by Illumina. Single-stranded DNA template for PCR was synthesized from the same RNA samples that were used for hybridization to the Affymetrix GeneChips using SuperScript First-Strand Synthesis System for RT-PCR (Invitrogen). For each genotype, we combined 1 μg of RNA from each of the six tissue types and converted it to a single-stranded cDNA according to the manufacturer's recommendations using oligo(dT)_12-18 _as a primer. Single-stranded DNA was diluted fivefold and 2 μl was used for PCR amplification using gene-specific primers and HotStart Taq polymerase (Qiagen) with the following thermocycling parameters: 15 min 95°C, followed by 40 cycles of 30 sec 95°C, 45 s 60°C and 2 min 72°C, with a 10 min final extension at 72°C. PCR products were treated with ExoSAP-IT reagent (USB Corporation) and sequenced with the same primers using BigDye Terminator v3.1 cycle sequencing kit on an ABI PRISM 3700 sequencer (Applied Biosystems). Base-calling of ABI chromatograms and assembly of each unigene were done using Mutation Surveyor software (SoftGenetics, State College, PA). Synthetic chromatograms generated for all probe and EST unigene sequences were included in assemblies for comparison. Polymorphisms were called using Mutation Surveyor software and examined manually. SNP positions were recorded symmetrically, that is, a SNP in the central nucleotide of a 25-mer was in position 13, while SNPs in either first or twenty-fifth position was assigned position 1. Probes with multiple SNPs were allocated to a single group (Figure 3). Insertions and deletions were scored as polymorphisms, but the positions of polymorphisms were not scored.

### SNP discovery in a random EST contig set

An SNP discovery project is currently underway in our laboratory which is based on resequencing alleles of barley genes in a set of eight barley lines, including Morex and Golden Promise [30]. The same EST unigene assembly that was used to design the Affymetrix Barley1 GeneChip was used in this SNP discovery study; PCR was carried out on genomic DNA templates, however. The Morex and Golden Promise sequences were reassembled separately as described for the SFP sequence set. Three hundred contigs representing essentially a random sample without any prior knowledge of polymorphisms were selected from this set on the basis that they included sequences from both genotypes; did not contain introns; sequences from both genotypes covered at least six Affymetrix Barley1 GeneChip probes for each probe set.

## Figures and Tables

**Table 1:** SFP false discovery rate (FDR) estimates in RNA and genomic DNA hybridization data

RNA hybridization: 17 Golden Promise 19 Morex, 6 tissues; SAM analysis for the two-class unpaired case assuming unequal variances; s0 = 0.0342 (the 5% quantile of the s values); number of permutations, 500. Mean number of falsely called genes is computed.	Delta	p0	Called	False	FDR
	0.5	0.95	27,159	5,884	0.206
	1.0	0.95	17,744	594	0.032
	1.5	0.95	13,285	65	0.005
	2.0	0.95	10,504	7	0.001
	2.5	0.95	8,583	0	0.000
					
Genomic DNA hybridization three replicates three genotypes; SAM analysis for the multi-class case with three classes; s0 = 0.0123 (the 25 % quantile of the s values); number of permutations: 100; mean number of falsely called genes is computed.	Delta	p0	Called	False	FDR
	1	0.95	4,017	2,073	0.47
	2	0.95	1,728	583	0.31
	3	0.95	1,090	258	0.22
	4	0.95	789	139	0.16
	5	0.95	631	86	0.13

The Bioconductor package siggenes [37,36] was used to derive SFP calls at various thresholds in the original data and randomly permuted data according to SAM [39]. Delta, the threshold; p0, the prior probability of the proportion of SFP in the null dataset; Called, the number of SFP at each threshold; False, the number of SFP in the mean permuted dataset.

**Table 2:** Single feature polymorphism (SFP) comparison with sequence-characterized SNPs

		GeneChip		
		
		mxSFP	nonSFP	gpSFP
RNA sequence		5,301	240,307	5,203
MX	178	115	45	18
Non-polymorphic	2,200	27	2,045	128
GP	223	7	61	155
				
Chi-square = 2,049.2, df = 4, *p*-value = 0				

The categories for SFP calls from RNA data are shown in columns: mxSFP, SFP in Morex; nonSFP, no SFP at the 0.1% FDR; gpSFP, SFP in Golden Promise. The categories of sequence-characterized probes are in rows: MX, polymorphism in Morex; non-polymorphic, no polymorphism between probe and any of the two genotypes; GP, polymorphism in Golden Promise. Intersections of the columns and rows indicate different combinations of sequence-verified polymorphisms and SFP.

**Table 3:** SFP discovery in individual tissue types

Tissue	ALL	COL	CRO	GEM	LEA	RAD	ROO
Replicates (GP, MX)	18,18	3, 3	3, 3	3, 3	3, 3	3, 3	2, 4
Sensitivity	67%	52%	58%	63%	51%	62%	60%
False sequence polymorphism rate	40%	35%	34%	34%	34%	34%	35%
% variance explained	38%	30%	33%	37%	32%	31%	34%

Replicates indicate the number of arrays from each genotype analyzed for a given tissue type. Sensitivity is a percentage of correctly predicted SFP (270; Table 2) from the number of known sequence polymorphisms (401; Table 2). False sequence polymorphism rate is the percentage of predicted SFP that were found not to contain a DNA base-pair change. The % variance explained is that from a linear model fit of genotype (-1:MX; 0: no polymorphism; 1:GP) versus SFP d-statistic.

**Table 4:** Comparison of SFP prediction in RNA and genomic DNA hybridizations

		GeneChip RNA	
		
		SFPs	nonSFPs
GeneChip gDNA		10,504	240,307
SFPs	1,090	114	976
nonSFPs	24,9721	10,390	239,331
			
Chi-square = 107.28, df = 1, *p*-value = 3.863e-25			

SFP and non-SFP probes in the gene-expression data are in columns, while the genomic data are in rows.
